# The willingness and influencing factors to choose institutional elder care among rural elderly: an empirical analysis based on the survey data of Shandong Province

**DOI:** 10.1186/s12877-023-04615-5

**Published:** 2024-01-04

**Authors:** Xinglong Xu, Peiyao Li, Sabina Ampon-Wireko

**Affiliations:** School of Management, Jiangsu University, 301 Xuefu Road, Zhenjiang, Jiangsu P. R. China

**Keywords:** Institutional elder care, Willingness to accept institutional elder care, Population Aging

## Abstract

**Background:**

The ageing of the population has become an escalating problem in China, which has led to an increasing demand for healthcare throughout society. The care services of elderly institutions, as a more mature way of aging, can alleviate various social problems brought about by ageing to a certain extent. The aim of this paper is to explore the degree of acceptance of institutional care by rural elderly people in Shandong Province and the factors that influence whether rural elderly people accept institutional care services.

**Methodology:**

Based on the theory of planned behavior, an analytical framework was constructed for the willingness of rural elderly people to receive nursing services from elderly care institutions. Using survey data from 192 rural elderly people in Shandong Province, descriptive statistics, binary logistic regression, and horizontal comparative analysis methods were used to analyze the willingness of rural elderly people to provide for the elderly and its influencing factors.

**Result:**

Only 17.71% of respondents expressed willingness to receive services from elderly care institutions. Among them, education level, trust in elderly care institutions, and support from adult children have a significant positive impact on whether rural elderly people receive nursing services from elderly care institutions; The number of children, the level of understanding of elderly care institutions, neighbors' choices of elderly care methods, and their ability to contribute to the family have a significant negative impact on whether rural elderly people receive nursing services from elderly care institutions. There are significant differences in the willingness and influencing factors of rural elderly people to provide for the elderly among different regions.

**Conclusion:**

The non-acceptance of institutional care by rural older people is a general phenomenon rather than a sample characteristic, thus justifying the supplementary status of institutional care services. The pension intention of the rural elderly in Shandong Province is obviously affected by personal will factors, and the influencing factors are various. The traditional concept of old-age care in Shandong province has a strong path-dependent effect on the choice of the rural elderly. There is heterogeneity in the willingness and influencing factors of the rural elderly in different regions and countries. Based on this, this paper puts forward the following suggestions: strengthen the spiritual and cultural construction of residents; The government should pay attention to the correct guidance of public opinion; And increase pension subsidies. It is hoped that reduce the burden of national elderly care through these suggestions.

## Introduction

According to the data of China's seventh national census, the proportion of the population aged 65 and above has reached 13.50%, and the degree of population aging has been higher than the international standard of "aging society", and the burden of Chinese society's pension pressure is increasing. In this context, how to achieve " Old people have old-age security " has become the focus of concern of the government and academia. However, due to economic conditions, physical fitness and other factors, it is difficult for the elderly to transform their demand for medical services into accessible medical services in recent years. Especially in rural areas of China, this problem has a great negative impact on the health and quality of life of the rural elderly, and makes the problem of rural pension in China more serious and urgent [1]. Therefore, institutional elder care, as a socialized endowment model, is an important supplement to family endowment and an inevitable trend in the development of China's endowment model.

Although the willingness of the rural elderly to support themselves is diversified, most of them still choose the family pension mode, and the willingness to live in institutional elder care is low. The investigation of scholars can also prove this phenomenon. For example, Liu et al. [2] conducted a survey on the pension intention of 517 rural elderly in Changde City, Hunan Province, China, and found that 78.3% of the elderly were willing to accept family pension, and only 10.8% of the elderly were willing to accept nursing services in institutional elder care. However, in the real environment, there are many and complex factors that affect the willingness of rural elderly people to accept nursing services in institutional elder care. Simonson et al. [3] focuses on economic factors and finds that the elderly with better economic conditions tend to choose institutional care services. Williams et al. [4] conducted a study based on the individual characteristics of the elderly and found that the lower the physical fitness level and health status, the stronger the willingness to accept the nursing service of the nursing institution.

In general, the research on institutional elder care intention has made a lot of achievements, but the research results are not uniform, and most of the existing research analyzes its influencing factors from the objective conditions, namely individual characteristics, and ignores people's will as an important variable determining their behavioral intention. At the same time, most of the research objects are the urban elderly. The pursuit of happy life in the old age of the rural elderly is ignored, which is not conducive to fully explaining the realistic obstacles that affect the pension intention of the social elderly group.

Therefore, based on the theory of planned behavior, this paper will conduct a field survey on whether the elderly accept nursing services in institutional elder care. This paper focuses on the will factors that affect the willingness of the rural elderly to accept nursing services in institutional elder care in Shandong Province, and finds the key factors that restrict the willingness of the rural elderly to accept nursing services in institutional elder care, so as to lay a research foundation for improving the willingness of the rural elderly to accept nursing services in institutional elder care and alleviating the severe pension situation in rural Shandong Province.

The structure of this paper is as follows: the first part is the introduction, introducing the research background and significance of this paper; The second part is the analysis of the influence mechanism, which mainly includes the theoretical basis, index system and theoretical analysis framework construction; The third part is the research hypothesis; The fourth part is the research design, which mainly introduces the data sources, variable assignment and research methods of this paper. The fifth part is empirical analysis, and the robustness of the model estimation is tested by the method of endogeneity diagnosis. At the same time, the horizontal comparison method was used to analyze the differences of pension intention in different regions. The sixth part is the research conclusion; The seventh part discusses the limitations of this study.

## Influence mechanism analysis

### Theoretical basis

The theory of planned behavior is considered as one of the typical theories for predicting consumer behavior, which has strong predictive and explanatory power [5]. The main idea of the theory of planned behavior [6] is that behavioral attitude, subjective norm and perceived behavioral control jointly affect behavioral intention, and behavioral intention determines the final actual behavior. Among them, behavioral attitude refers to the positive or negative feelings that individua ls hold about a particular behavior. Subjective norms refer to the social group pressure that individuals feel on whether to adopt a specific behavior, that is, people are affected by the views of important people (mainly refers to their relatives, organizations, neighbors and friends or people who have great influence on them) on this specific behavior [7]. Perceived behavioral control refers to the obstacles that reflect the individual's past experience and expectations, that is, whether they have the conditions to take actual actions. Behavioral attitudes, subjective norms, and perceived behavioral control are collectively referred to as volitional. Behavioral intention refers to the actual intention to do a particular behavior. In addition, individual characteristics (such as age, gender, education level, etc.) also indirectly affect behavioral intention by influencing behavioral attitudes, subjective norms, and perceived behavioral control.

### Construction of index system

The influencing factors selected in this paper are shown in Table 1. Behavioral and attitude variables include: (1) the degree of trust in nursing home institutions (whether to trust the service items and quality provided by nursing home institutions) and (2) the degree of understanding of nursing home institutions. That is, the degree of cognition of the respondents to nursing homes. These two indicators were selected to explore the attitudes of middle aged and elderly people in rural areas towards living in nursing homes. The subjective normative variables include: (1) the degree of influence of children's attitude on them; And (2) the degree to which they are influenced by their neighbors' choices. In a group-oriented country like China, individual behavior intentions are easily affected by group pressure [8]. The variables of perceived behavior control include: (1) the degree of commitment to the fees of the nursing home, (2) the degree of contribution of waste heat to the family (i.e., the frequency of home labor such as farm work, housework, and child care). These two indicators were selected to explore the practical feasibility of rural elderly people living in pension institutions. Individual characteristics were selected as gender, age, education level, occupation and number of children.

**Table 1:** Index system selection

Variables	Description
Attitude	The level of trust in the nursing home
	The level of knowledge about the nursing home
Subjective normative variables	The extent to which their children's attitudes affect them
	The extent to which the choices of their neighbors affect them
Perceptual behavioral control variables	The degree of commitment to nursing home fees
	The extent to which they contribute excess heat to the family

### Theoretical analysis framework

**Fig. 1:**
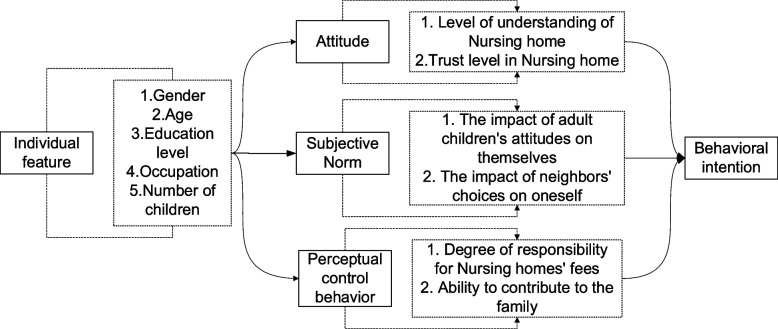
Theoretical analysis framework

Behavioral attitude, subjective norm and perceived behavioral control are three dimensions that determine behavioral intention of rural elderly people, and behavioral intention ultimately determines behavior. In addition, individual characteristics indirectly affect behavioral intention and behavior by influencing behavioral attitude, subjective norm and perceived behavioral control. Based on the theory of planned behavior, the analysis framework of this paper is constructed, as shown in Fig. 1.

## Research hypothesis

Based on the above theoretical basis and the construction of the influencing factors index system, this paper puts forward the following research hypotheses.

### Research hypothesis of individual characteristics

Personal characteristics have an important impact on the behavioral intention of accepting nursing home services. The existing studies on personal characteristics mainly include gender, age, education level, personal occupation factors and so on. Previous studies have shown that education level, occupation and the number of children have a significant impact on the elderly's choice of pension mode [9]. Therefore, this paper will focus on three factors of the elderly: education level, occupation and the number of children. Education level is the simplest way to measure the cognitive ability and thinking level of the elderly [10]. The education level of the elderly may influence their scientific knowledge of nursing homes. Meanwhile, the occupation of the elderly plays an important role in the decision-making process of whether to choose a nursing home. The occupational nature of work can affect the choice and traditional cognition of the elderly [4]. For example, the elderly engaged in professional work in enterprises and national institutions are affected by the working environment, and their ideological level and the degree of acceptance of new things are high. Therefore, they can timely, fully and correctly recognize the necessity and rationality of national policies, and can correctly and objectively view the nursing services provided by pension institutions, and they are more likely to choose pension institutions. In addition, in the traditional way of home care, family care is an essential and important factor. However, due to the reduction of employment opportunities in rural areas, a large number of young and middle-aged people go out to work, resulting in a reduction in the resources required for family pension. Most of the care support and emotional comfort that the elderly need in their later years come from friends, spouses, children, and even family networks [11]. When these needs are difficult to meet, the need for institutional care increases. Therefore, the more children rural elderly people have, the more likely they are to be cared for by their families, reducing the need for institutional care.

On this basis, the following hypotheses are proposed regarding the influence of individual characteristics on the willingness of rural elderly people to accept nursing sevices in pension institutions:

- H1: Individual characteristics have an impact on rural elderly people's acceptance of nursing services in pension institutions:
- H1a: The higher the education level of the elderly, the higher the possibility of choosing institutional care services;
- H1b: The easier the elderly are to be exposed to new things at work, the more willing 181 they are to accept the nursing services of nursing institutions;
- H1c: The fewer children the elderly have, the more likely they are to choose institutional nursing services.

### Research hypothesis of behavior and attitude

Behavioral attitude mainly refers to the positive or negative subjective feelings of the elderly towards the nursing service of the aged care institutions. The perception of the service content of the elderly for the aged was measured by their understanding of the elderly for the aged. The more the elderly know about the location, price, service content and other aspects of pension institutions, the more they can determine whether their old life can be fully protected and respected, so as to make the best choice according to their actual situation [12]. In reality, due to the two-way support of the government and social capital, China's pension institutions are developing in a good situation, and the facilities, staffing and service level in the institutions are steadily improving [13]. Therefore, when the rural elderly have an objective and clear understanding of the current status of nursing services in pension institutions, they tend to choose nursing services in pension institutions [14]. In addition, the degree of trust in pension institutions can directly reflect the likes and dislikes of rural elderly people to pension institutions. When the elderly maintain a supportive and trusting attitude towards the service content, service attitude and management system of the elderly care institutions, they tend to choose the nursing service of the elderly care institutions [15].

Based on this, the following research hypotheses are proposed in this paper:

- H2: Behavioral attitude influences rural elderly people's acceptance of institutional nursing services for the aged:
- H2a: The higher the knowledge level of the elderly about the nursing home, the higher the possibility of the elderly to choose the nursing home;
- H2b: The stronger the trust of the elderly in the nursing home, the higher the possibility of the elderly to choose the nursing home.

### Research hypothesis of subjective normative factors

Under the influence of China's family culture that attaches great importance to kinship, the willingness of rural elderly to accept nursing services in nursing homes is not only affected by behavior and attitude, but also greatly affected by family factors. Xie and Wang [16] summed up three social participation modes of the elderly in China: high participation mode, low participation mode and family care mode by comprehensively measuring the economic participation, political participation, public participation and family participation of the elderly. The family old-age care model is a unique model for the elderly in China, which has not yet appeared in Western studies. It can be seen that the willingness of the rural elderly to accept nursing services in pension institutions will also face obstacles from family characteristics. In fact, China's old-age care culture and tradition are unique. In the local context, the topic of old-age care cannot be separated from the "family" [17]. The "responsibility ethics" in China's old-age culture requires children to "feedback" to their parents, but parents' understanding of their children's life pressure can have a positive impact on their children to get rid of the shackle of traditional concepts, which is also part of China's traditional family concept. That is, parents' love for their children is far higher than all external constraints [18]. Therefore, when the burden of children's life is increasing and they have a supportive attitude towards their parents entering the nursing home, parents will choose to listen to their children because they understand the pressure of their children's life, so the possibility of choosing institutional care services increases. In addition to family factors, on the other hand, the subjective willingness of the rural elderly to choose nursing services in pension institutions will fluctuate under the influence of the external environment. Previous studies have shown that the subjective normative factors that affect the elderly's acceptance of institutional care services usually include the influence of external public opinion and social choice orientation [19]. In order to blend in with the local environment and culture and not be criticized by the public, the rural elderly tend to make the same choice as the public. Therefore, the social acceptance of nursing services in nursing homes also affects the rural elderly's views on nursing services in nursing homes [16]. When most people reject the nursing service of pension institutions, the rural elderly will subconsciously make a negative evaluation of the nursing service of pension institutions. However, when more and more people choose to accept nursing services in nursing homes, the rural elderly will re-evaluate the feasibility of nursing services in nursing homes, and the possibility of choosing nursing services in nursing homes will also increase.

Based on this, the following research hypotheses are proposed in this paper:

- H3: Subjective normative factors will affect rural elderly people's acceptance of nursing services in pension institutions:
- H3a: when children have a supportive attitude towards their parents living in nursing homes, the elderly are more likely to choose institutional nursing services;
- H3b: when the rural elderly are less affected by the choice of neighbors, the rural elderly are more likely to accept nursing services in nursing homes.

### Perceived behavioral control factors

Nursing homes tend to place financial requirements on service users [20]. Compared with the rural elderly with poor family economic conditions, the elderly with higher annual income or higher socioeconomic status are more likely to bear the cost of nursing services in nursing homes, so they are more likely to choose institutional nursing services. In addition to the limitation of economic factors, whether the rural elderly can continue to contribute to their families also affects their willingness to choose institutional care. Under the influence of the traditional Chinese family concept, the elderly always want to reduce the economic or spiritual burden for their children [21]. Therefore, when the elderly can reduce the family burden by doing housework, taking care of children or earning living expenses, they often do not choose nursing services in nursing homes. In addition, older people seek to realize their own value in their later years [22]. With the increase of age, the behavior of the elderly is gradually limited. Even under this condition, the elderly still want to realize their value pursuit by contributing surplus value. Therefore, when the elderly can relieve the burden of the family through their own ability, on the one hand, it satisfies the restriction of the traditional concept, on the other hand, it satisfies the needs of the elderly to realize their own value. At th is time, the possibility of the elderly to choose institutional nursing services for the aged is low.

Based on this, this paper puts forward the following hypotheses:

- H4: Perceived behavioral control factors will affect rural elderly people's acceptance of nursing services in pension institutions:
- H4a: The higher the degree of charge commitment, the higher the possibility of the elderly to choose institutional care services;
- H4b: The stronger the ability to contribute waste heat to the family, the less likely the rural elderly are to choose institutional nursing services for the aged.

## Study design

### Data sources

The data in this paper were derived from the questionnaire survey of "institutional pension intention" of 204 rural elderly people in three counties of Shandong Province from April to June 2022. The survey was organized by the members of the research group. According to the socio-economic development of the counties and townships surveyed, the survey samples were selected by multi-stage probability sampling method. The reason why Shandong rural area was selected as the survey site and stratified sampling was carried out according to the ladder type differences in the middle, east and west is based on the following two considerations: First, Shandong's economy continues to develop, family size continues to shrink, aging is aggravated, and the number of empty-nest elderly increases sharply, which is consistent with the status quo of most rural areas at home and abroad, so the region has a certain representative. At the same time, Shandong is deeply influenced by Confucianism and has certain particularness, which can provide new ideas for domestic and foreign scholars to study the pension intention of the elderly. Second, due to the geographical location, Shandong's social development and pension services show significant regional differences in the form of ladders in the middle, east and west regions. Therefore, a comparative study of these three regions to clarify their common points and differences can more carefully and clearly investigate the specific influencing factors of the development of rural elderly people's willingness to accept nursing services in pension institutions. To provide empirical support for improving relevant policies.

**Fig. 2:**
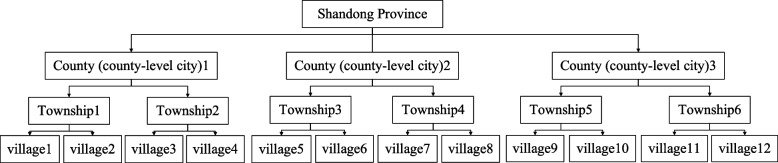
Sampling flow chart

The survey population in this study is residents aged 60 years and above who have rural household registration and live in rural areas in 78 counties (county-level cities) under the jurisdiction of Shandong Province. According to the method of multi-stage probability sampling, the actual sampling of survey samples is carried out: Firstly, 78 counties (county-level cities) under the jurisdiction of Shandong Province were used as sampling frame, and the county (county-level city) was used as sampling unit, and one county (county-level city) was selected from the middle, east and west regions of Shandong Province by stratified sampling method. Secondly, two towns were randomly selected from the selected counties (county-level cities). Thirdly, two villages were randomly selected from the selected towns. Finally, 17 elderly people were randomly selected from the selected villages as the subjects of the questionnaire survey. Considering the possibility that some elderly people are not convenient to read and fill out the questionnaire in person due to physiological conditions or educational level limitations, this survey adopted the method of filling out the questionnaire on behalf of the rural elderly to carry out an empirical study on whether the rural elderly will accept nursing services in nursing homes. A total of 204 questionnaires were distributed and 204 questionnaires were collected. After excluding the samples with missing values, the effective sample size was 192. The specific sampling process is shown in Fig. 2.

Due to the ethical issues involved in the investigation process, as the survey did not involve any private information of the respondents, we obtained their verbal consent and expressed their willingness to participate in this elderly care willingness survey.

### Variables and measurement

In this study, the dependent variable was the willingness to accept nursing services in elderly care institutions, and the independent variables were divided into four dimensions: behavioral attitude, subjective normative factors, perceived behavioral control factors, and individual characteristics. Each dimension was measured by more specific items. The specific variable assignment is shown in Table 2.

**Table 2:** Variable assignment

Variables	Dimensions	topic	Assignment
Dependent variables	Intention to retire	Willingness to accept nursing service in nursing home	Yes = 1; No = 0
Independent variable	Attitude	The level of trust in the nursing home	Trust = 3; Average = 2; Distrust = 1
		The level of knowledge about the nursing home	Understanding = 3; Average = 2; Do not know = 1
	Subjective norm factor	The extent to which their children's attitudes affect them	Supportive = 3; Average = 2; No support = 1
		The extent to which the choices of their neighbors affect them	Impact = 3; Average = 2;Not influential = 1
	Perceived behavioral control	The degree of commitment to nursing home fees	Strong = 3; Average = 2; Weak = 1
		The extent to which they contribute excess heat to the family	Strong = 3; Average = 2; Weak = 1
	Individual characteristics	Gender	Male = 1; Female = 2
		Age	60 ~ 74 years = 1; 75 ~ 89 years = 2; ≥ 90 years = 3
		Level of education	Primary school and below = 1; Secondary = 2; High school = 3; College or higher = 4
		Career	Agriculture = 1; Labor = 2; Enterprises and institutions = 3; Self-employed = 4
		Number of children	None = 0;1 or more = 2;3or more = 3

Among them, the degree of trust in pension institutions, the degree of understanding of pension institutions, the degree of influence of adult children's attitude on the willingness of rural elderly people to care for the elderly, and the degree of influence of neighbors' choice on the willingness to care for the elderly are divided into three levels from strong to weak. Compared with the average annual income of the elderly and the local pension institutions, the degree of commitment of the elderly in rural areas is weak when the annual income of the elderly is 50% or more, the affordability is strong when the income of the elderly is 20% or less, and the affordability is average when the proportion is > 20% and < 50%. The ability to contribute waste heat to the family is measured by the mental and physical state of the elderly. When the elderly's mental health is normal, the body has no disease or the disease does not affect the normal life, the ability to contribute waste heat is judged to be strong. When the elderly have a sense of autonomy, but chronic diseases and other diseases indirectly affect the normal life of the elderly, it is judged that the ability to contribute waste heat to the family is average; When the elderly lose their autonomous consciousness, the physical disease affects their daily life and even brings spiritual or material burden to the family, it is judged that the ability to contribute waste heat to the family is poor. In terms of individual characteristics, the occupation of the elderly is mainly judged as the main work they engaged in before retirement. If they engaged in agricultural cultivation for a long time to obtain material resources, they are judged as farming. If he has worked in light industry, heavy industry and other enterprises for a long time to obtain income, he shall be judged as a migrant worker; If you earn income by starting your own business or setting up a stall, you are judged to be self-employed; If you work in a state institution or a government department, you are judged to be an enterprise or institution.

### Statistical methods

The dependent variable in this paper is the willingness of the rural elderly to provide for the elderly in institutions, which is a binary variable, and is suitable for the use of binary logistic regression model. Let the dependent variable be y, with a value of 1 indicating that the rural elderly are willing to choose institutional pension, and a value of 0 indicating that the rural elderly are not willing to choose institutional pension. The *m* independent variables affecting* y* are recorded as x_*1*_*, x*_*2*_*, ……, x*_*m*_*.* Control variables are *C*_*1*_*, C*_*2*_*, ……, C*_*k*_. Suppose that the conditional probability of rural elderly people willing to choose institutional endowment is *p (y* = *1/x)* = *p*_*i*_, and *1-p*_*i*_ represents the probability of rural elderly people unwilling to choose institutional endowment.

$${p}_{i}=\frac{1}{1+{e}^{-(\alpha +\sum_{i=1}^{m}{\beta }_{i}{x}_{i}+\sum_{k=1}^{m}{\beta }_{k}{c}_{k})}}=\frac{{e}^{\alpha +\sum_{i=1}^{m}{\beta }_{i}{x}_{i}+\sum_{k=1}^{m}{\beta }_{k}{c}_{k}}}{1+{e}^{\alpha +\sum_{i=1}^{m}{\beta }_{i}{x}_{i}+\sum_{k=1}^{m}{\beta }_{k}{c}_{k}}}$$

The probability ratio *p*_*i*_*/(1-p*_*i*_*)* of the rural elderly who are willing to choose institutional pension and who are not willing to choose institutional pension is called the event occurrence ratio, abbreviated as "Odds". The odds is positive (because *0* < *pi* < *1*) and has no upper bound. Perform logarithmic transformation on Odds to obtain the linear expression of the logistic regression model:

$${\text{ln}}\left(\frac{{p}_{i}}{1-{p}_{i}}\right)=\alpha +\sum_{i=1}^{m}{\beta }_{i}{x}_{i}+\sum_{k=1}^{m}{\beta }_{k}{c}_{k}$$

In the first and second formulas, *α* Is a constant term; *m* is the number of independent variables; *β*_*i*_ is the coefficient of the independent variable, *β*_*K*_ is the coefficient of the control variable, reflecting the influence direction and degree of the independent variable on the willingness of the rural elderly to provide for the elderly in institutions.

## Results and analysis

### The willingness level of the rural elderly to provide for the elderly in institutions

According to the analysis results in Table 3, 17.71% of the elderly are willing to accept the nursing service of the nursing home, and 82.29% of the rural elderly are unwilling to accept the nursing service of the nursing home, indicating that the majority of the rural elderly have little motivation to choose institutional pension. From the perspective of the willingness of rural elderly of different sexes, there are certain differences between men and women, but the difference is very small.

**Table 3:** The willingness of rural elderly of different gender groups to provide for the aged

Willingness to provide for the aged		Gender grouping		Total
		Male	Female	
Would you choose to spend your old age in a nursing home?	Yes(%)	9.37	8.34	17.71
	No(%)	31.25	51.04	82.29

Pearson chi-square value = 2.598, *P* = 0.107

Moreover, the chi-square test results (*P* = 0.107) show that this difference does not exist in the whole population. In other words, the reluctance of rural elderly people to accept nursing services in nursing homes is not only a sample feature but also a common phenomenon. The inspiration from this is that in rural areas, it is reasonable to position institutional pension as a supplementary position in the pension service system, at least this is more in line with the wishes of the current rural elderly.

### Influencing factors of the willingness of the rural elderly to care for the aged in institutions

In order to ensure the reliability of the questionnaire data, Cronbach's coefficient method was used to measure the reliability of the questionnaire data, and the result was α = 0.754. KMO test and Bartlett's spherical test were used to test the validity of the questionnaire data, KMO = 0.687, Barlett's spherical test showed that the difference was statistically significant (χ2 = 659.445, *P* < 0.001). Therefore, the survey data used in this paper has certain reliability and can reflect certain problems.

In order to avoid possible multicollinearity among independent variables and to more clearly reflect the differences in the influence of independent variables of different dimensions on the rural elderly's acceptance of nursing services in pension institutions, this paper uses the stepwise regression method. To analyze the influence of individual characteristics (equation 1), behavior attitude (equation 2), subjective norms (equation 3) and perceived behavioral control (equation 4) on the rural elderly's acceptance of nursing services in pension institutions. The results of the analysis are shown in Table 4.

**Table 4:** Logistics analysis results of the willingness of rural elderly to accept nursing services in nursing homes

Dimension	Items	Equation (1)		Equation (2)		Equation (3)		Equation (4)	
		β	Exp(β)	β	Exp(β)	β	Exp(β)	β	Exp(β)
Individual characteristics	Gender	-0.219	0.178	-0.090	0.310	-0.085	0.087	-0.081	0.106
	Age	-0.022	0.580	0.043	0.574	0.009	0.200	-0.051	0.304
	Level of education	0.470**	3.038	0.177**	2.231	0.184**	4.906	0.273**	5.126
	Career	-0.050	0.747	-0.006	0.858	-0.012	0.600	-0.038	0.868
	Number of children	-0.223**	0.533	-0.126**	0.089	-0.055*	0.916	-0.072*	0.446
Behavior attitude	The level of knowledge about the nursing home			-0.222**	0.345	-0.182**	0.260	-0.095**	0.280
	The level of trust in the nursing home			0.483**	2.106	0.368**	1.994	0.327**	1.596
Subjective normative variables	The extent to which their children's attitudes affect them					0.213**	1.153	0.251**	1.697
	The extent to which the choices of their neighbors affect them					-0.187**	0.430	-0.129**	0.579
Perceptual control variables	The degree of commitment to nursing home fees							0.121*	1.521
	The extent to which they contribute excess heat to the family							-0.015**	0.628
Model fit effect	Log likelihood	117.425		56.900		55.800		77.123	
	Cox&Snell	0.276		0.471		0.474		0.413	
	Nagelkerke	0.454		0.777		0.782		0.680	

Note: "*, **" represent the significance of the variable at the 10% and 5% levels

According to the analysis results in Table 4, individual characteristics, behavioral attitudes, subjective normative factors, and perceived control behavior all have different degrees of significant influence on the willingness of rural elderly people in Shandong Province to accept nursing services in pension institutions. Among them, equation 1, equation 2, equation 3 and equation explained 45.4%, 77.7%, 78.2% and 68.0% of the changes in the willingness of the rural elderly in Shandong Province to accept nursing services in pension institutions, respectively. Specifically, there are:

Firstly, the influence of individual characteristics on whether rural elderly people accept nursing services in nursing homes. Among the individual characteristics, only edu-cation level and the number of children owned by the elderly had a significant impact on the willingness of rural elderly to accept nursing services in pension institutions. Among them, the degree of education has a positive impact on it, that is, the rural elderly with a higher education level are more likely to accept the nursing service of pension institutions. This result is similar to the research results of Zhang et al. [23]. The possible explanation is that the higher the education level of the elderly, the more opportunities they have to accept new things and the more open-minded they are, which also enables them to have an objective understanding of the quality and innovation of nursing services in nursing homes, and the higher their acceptance of nursing services in nursing homes. The number of children owned by the elderly has a negative impact on whether the rural elderly accept nursing services in pension institutions. That is to say, the more children the elderly have, the easier they are to accept the nursing service of pension institutions. This is similar to the findings of Michie et al. [24]. Compared with the rural elderly with few children, a large number of children means that the elderly have more family, friends and even community network resources, which can effectively meet the needs of the elderly for spiritual com- fort, alleviate the loneliness in their later years, and thus reduce the needs of the elderly for institutional care. Different from previous studies, occupation did not have a signifi- cant effect on different groups of rural elderly. Zhao and Li [25] found in the investi- gation of the elderly migrant workers that the experience of migrant workers can signifi- cantly promote the transformation of rural pension concepts from traditional to modern, but for the returning elderly who have been migrant workers for less than 10 years, the experience of migrant workers does not change the traditional family pension concept. And still cannot accept the social way of old-age care. It shows that in a short period of working outside the city, the living experience and contact with modern values are not enough to compete with the traditional concept of farmers, and the elderly are still used to making judgments and choices with traditional thinking. Therefore, even if the rural elderly have the opportunity to come into contact with new things in their work, it is dif- ficult to change the deep-rooted traditional ideas in people's hearts due to the limitation of working hours. It can be seen that, as the historical accumulation of traditional agricul- tural civilization, China's traditional pension concept has a strong path-dependent effect. Therefore, H1a and H1c have passed the hypothesis verification, while H1b has failed.

Secondly, the influence of behavioral attitude on whether rural elderly people accept nursing services in nursing homes. In the behavioral attitude, the degree of trust in nurs-ing home institutions has a significant positive impact on the willingness of the elderly to accept institutional care, which is consistent with the study results of Sherman Folland [26]. However, the knowledge of nursing home institutions has a significant negative effect on the willingness of the elderly to live in nursing homes, which is inconsistent with the statistical results of Liu et al. [27]. The possible explanation is that Shandong Province, as the birthplace of Confucianism, has a deep-rooted traditional concept of old-age care. Under the intervention of public opinion, the elderly will also have a sense of shame and insecurity when they live in nursing homes. This psychological state can also affect the subjective judgment of the elderly. Secondly, the development of elderly care institutions in rural areas of Shandong province is still in its infancy. When the service level and quality of pension institutions fail to meet the psychological expectations of the elderly and their pension needs, the willingness of the rural elderly to accept the service of pension institutions will decline [28]. Therefore, H2b passed the hypothesis test, and H2a failed.

Thirdly, the influence of subjective normative factors on whether the rural elderly accept nursing services in nursing homes. Among the subjective normative factors, the degree of support from adult children has a positive correlation with whether the rural elderly accept nursing services in nursing homes. As Chen and Ye [29] pointed out in their study, the elderly's understanding of new things is limited. When adult children continue to export objective information about nursing homes to the elderly and maintain a supportive attitude towards nursing home admission, the elderly will make clearer and more rational choices and therefore are more inclined to stay in nursing homes. The degree of influence of neighbors' choice of pension methods has a negative correlation with whether the rural elderly accept nursing service in nursing homes. This indicates that the stronger the influence of neighbors' choice of pension mode on the rural elderly, the lower the possibility of the elderly to choose nursing services in pension institutions. The possible reason is that the older people who are more strongly influenced by their neighbor's choice have more obvious herd mentality [30]. At present, the Chinese society's acceptance of nursing services in pension institutions is still low, and the pressure of social opinion and the guidance of the external environment are the reasons. Therefore, it is easy to guide the rural elderly with a herd mentality to reject the nursing services of pension institutions. Therefore, H3a and H3b were verified.

Finally, the perceived behavioral control factor had a significant impact on whether the rural elderly accepted nursing services in nursing homes. In this dimension, the degree of contributing waste heat to the family has a negative correlation with the dependent variable. This is similar to the findings of Melanie and Sereny [22]. Possible reasons include: first, the influence of traditional Chinese concepts makes the elderly group want to do their best to help their children or family to reduce the burden. Second, the pursuit of self-worth. Compared with young people, the elderly prefer to reflect their own value in their later years rather than avoid self-denial and negative life attitudes [18]. Therefore, when the rural elderly have the ability to contribute to the family's waste heat, they are less likely to choose the nursing service of the aged care institution. The degree of payment of pension institutions has a positive correlation with whether the rural elderly accept the nursing service of pension institutions. This is consistent with the research results of Dong Xiaoqiang [31]. The reason may be that nursing homes have certain requirements for the economic ability of the elderly, but for families with low annual income, the annual income and pension reserve of the family are not enough to pay for institutional nursing costs. If the family only pays for nursing services, the family's economic risk bearing capacity will decline sharply. Therefore, the elderly who have a low commitment to the fees of nursing homes are less willing to accept the care of the elderly. Therefore, both H4a and H4b passed the verification.

### Robustness check

Robustness testing is an important factor in testing the quality of empirical results. The omission of important variables, reverse causality and other factors lead to the en-dogenous nature of the model, which is an important reason for the poor robustness of the estimation results [32]. There are many ways to test endogenous problems, such as variable transformation, using new analysis methods, adding new control variables, and using instrumental variables [33]. In order to test the robustness of the regression results, this paper added the control variable of family relationship, and replaced the original model with Robust model to test the stability of the model estimation results again.

According to the regression results in Table 5, although the degree of influence of different dependent variables on the dependent variable has changed in the final regression results, the direction of influence of the main independent variables on the dependent variable is basically the same as that of the original model. It can be seen that the estimation results in this paper are robust and reliable.

**Table 5:** Robustness test

Variables	New control Robust model variables	Robust model
Family ties(bad = 0)	0.044(1.577)	
Gender	-0.055(-1.054)	-0.052(-1.255)
Age	-0.033(-0.790)	-0.008(-0.247)
Career	-0.007(-0.185)	-0.012(-0.383)
Education level	0.073**(2.985)	0.078**(4.000)
Number of children	-0.028*(-1.018)	-0.040*(-1.902)
The level of knowledge about the nursing home	-0.039*(-1.201)	-0.060*(-2.325)
The level of trust in the nursing home	0.114**(3.518)	0.092**(3.611)
The extent to which their children's attitudes affect them	0.095**(2.826)	0.119**(-4.506)
The extent to which the choices of their neighbors affect them	-0.039*(-1.527)	-0.027*(1.341)
The extent to which they contribute excess heat to the family	-0.113**(-3.494)	-0.084**(3.292)
The degree of commitment to nursing home fees	0.065**(1.536)	0.004*(0.159)
R2	0.651	0.622
Adjust R2	0.603	0.575
F-value	13.542**	13.176**

Note: "*, **" represent the significance of the variable at the 10% and 5% levels

### Horizontal comparative analysis

In order to more comprehensively understand the heterogeneity of the influencing factors of the rural elderly's willingness to care for the aged in Shandong Province and other regions and countries, this paper will conduct a horizontal comparative analysis between different regions and countries.

Based on the existing data and research results, it is found that the overall willingness of the rural elderly in China to accept nursing services in nursing homes is low, and there is a large difference between different regions. The rural elderly in economically developed Beijing, Shanghai and Shenzhen tend to accept nursing services in nursing institutions, while the proportion of rural elderly in underdeveloped Henan, Heilongjiang and Guangxi receiving nursing services in nursing institutions is the lowest in China [34–36]. As developed countries, the United Kingdom, the United States and South Korea have a more serious degree of aging and a sharp increase in social pension pressure. In this context, different from the low proportion of the rural elderly in our country, the rural elderly in the United Kingdom, the United States and South Korea all show high willingness to care for the elderly in institutions [10, 28].

By comparing the influencing factors of the willingness of rural elderly people to accept nursing services in nursing homes in Shandong Province and other regions, it can be found that at the level of individual characteristics, the willingness of rural elderly people in developed and backward areas to accept nursing services in nursing homes is affected by education level and the number of children they have [17, 18]. Different from the results of this study, the occupation of rural elderly in economically developed areas such as Beijing, Shanghai, and Zhejiang Province has a significant impact on the willingness to accept nursing services in pension institutions, while the education level of rural elderly in developed countries such as the United Kingdom and South Korea has no significant impact on the willingness to care for the elderly [9, 28].

In terms of behavior and attitude, relevant research conclusions show that rural elderly people in Zhejiang, Guangdong, Shanghai and other places with relatively developed economies and well developed pension security systems have a deeper understanding of pension institutions, and are more inclined to accept nursing services in pension institutions [20, 37]. In western and some central areas of China, where the development of pension security system is relatively backward, the degree of understanding of pension institutions has a negative impact on the rural elderly's acceptance of nursing services in pension institutions. Different from the results of this study, rural elderly people in developed regions such as the United States and South Korea value the service quality and geographical location of nursing homes more than the influence of traditional concepts [3, 15].

In terms of subjective normative factors, adult children's support in different regions of China mostly has a positive correlation with the acceptance of nursing services by nursing institutions for the rural elderly [27, 38]. In contrast to the results of this study, rural elderly people in the United States and South Korea are almost unaffected by the choice of neighbors and are less influenced by social opinion [14, 39].

In terms of perceived behavior control factors, the same as the rural elderly in Shandong Province, the degree of rural elderly contributing to the waste heat of the family in Henan Province, Anhui Province, Hebei Province and other places was negatively correlated with whether the rural elderly accepted nursing services in pension institutions. However, Zhejiang Province, Beijing, Shanghai and Guangdong Province did not show significant influence on the factor of household waste heat contribution [40, 41]. Whether the rural elderly accept nursing services in nursing homes in other regions of China or other countries is affected by the degree of economic commitment, but the direction of its influence is slightly different [26, 42].

## Conclusions

Based on the above research, this paper draws the following conclusions:

1. It is a common phenomenon in China that the rural elderly are not willing to accept the nursing service of pension institutions. Combined with the data analyzed in this paper, among the 192 valid samples, 34 people had the intention of institutional care, accounting for 17.71% of the total sample. It can be seen that the willingness of rural elderly people in Shandong Province to accept nursing services in nursing homes is very low, and most people are not willing to choose nursing services in nursing homes.
2. Whether the rural elderly people in Shandong Province accept nursing service in nursing homes is obviously affected by personal will factors, and the influencing factors are diverse. The willingness of the rural elderly to accept nursing services in nursing homes in Shandong Province is obviously affected by individual will factors. Behavioral attitude, perceived behavior control factors and subjective norm factors all have a significant impact on the rural elderly in Shandong Province to accept nursing services in pension institutions.
3. The traditional concept of elderly care in Shandong province has a strong path dependence effect. The study found that pre-retirement occupation has no significant effect on the acceptance of nursing services in nursing homes for the rural elderly, while the degree of understanding of nursing homes has a significant negative correlation with the acceptance of nursing services in nursing homes for the rural elderly. It can be seen that the rural elderly in Shandong province are generally affected by the traditional concept of filial piety and old-age care, and the external environment and reality are difficult to resist the 568 traditional concept.
4. There are regional differences and characteristics in whether the rural elderly accept the nursing service in the aged care institutions.

The horizontal comparison found that the willingness of the rural elderly in China to accept nursing services in pension institutions is not high, but the willingness of the economically developed areas in China to accept nursing services in pension institutions is high. At the same time, the rural elderly in the developed countries such as the United Kingdom, the United States and South Korea have a high level of overall willingness to accept the nursing service of pension institutions. In terms of influencing factors, the ability to pay fees for pension institutions has an impact on the willingness of rural elderly at home and abroad. The difference is that the elderly in foreign countries pay more attention to the objective conditions such as the service quality and geographical location of pension institutions, while the willingness of the rural elderly in China is generally constrained by the social environment such as lifestyle and ideas.

The corresponding policy implications of this paper's conclusion are as follows:

1. Strengthen the construction of spiritual civilization and dilute the strong traditional concept of pension Strengthen the construction of spiritual civilization, actively encourage the rural middle aged and elderly people to open their minds and recognize the reality, and gradually make modern spiritual civilization deeply rooted in the hearts of the people, so that people really understand that living in nursing homes is not the behavior of children's unfilial piety, and will not bring adverse reputation to the family, but will reduce the burden of children's life and mental pressure [43]. More community-type nursing homes should be established, that is, nursing homes close to home, and children should be encouraged to visit their parents in nursing homes. In this way, the shackles of old ideas in the hearts of rural middle-aged and elderly people can be gradually unshackled, and at the same time, the pressure brought by groups in subjective normative factors on rural middle-aged and elderly people can be reduced, that is, the negative influence of children and neighbors can be reduced [21].
2. The government pays attention to the correct guidance of public opinion and corrects the cognition of rural elderly people on nursing homes Strengthen the construction of various hardware and software facilities such as food and services in modern nursing homes to improve the quality of life of residents in nursing homes [23]. Increase the publicity of modern nursing homes. The information communication channels in rural areas are mainly divided into online channels and offline channels. Online channels include radio, newspaper, television and mobile phone. Among the middle-aged and elderly people in rural areas, television is the most widely used channel, which can increase the propaganda of the construction of modern nursing institutions. Offline channels can lead local respected middle-aged and elderly people to visit nursing homes, bring the actual situation of modern nursing homes back to the village, and gradually modify the villagers' cognition of nursing homes [44].
3. Increase pension subsidies to reduce the economic pressure on the rural elderly to receive nursing services in nursing homes To reduce the difficulty of rural middle-aged and elderly people to bear the fees of pension institutions, we should accelerate the development of local economy and increase the pension subsidies. We should increase the development and support of rural economy, promote the development of rural finance, maximize the radiation of urban economy to rural areas, promote the development of local industrialization, and increase farmers' income. In addition to providing subsidies for the construction and operation of pension institutions, we should increase the subsidy for the occupancy cost of pension institutions, reduce the expenditure of pension institutions for farmers, reduce the proportion of pension institutions' expenses in farmers' income, and reduce the difficulty for rural middle aged and elderly people to bear the fees of pension institutions [37].

## Limitations of the study

Based on the theory of planned behavior, this paper focuses on analyzing the influence of individual will of the rural elderly on their willingness to accept nursing service in pension institutions. However, there are still some deficiencies in this study. For example, due to the limited ability of the authors to collect data on whether the rural elderly in Shandong Province accept the care services of nursing homes, it is not possible to comprehensively understand whether the rural elderly in Shandong Province accep the care services of nursing homes from the perspective of time, sample size and content. Secondly, due to the realistic environment, most of the respondents moved away from their original residence, and it was impossible to contact them, so it was difficult to carry out an intertemporal longitudinal study.

The author will continue to improve the existing research to understand more comprehensive pension needs, so as to make the empirical results more scientific and comprehensive, and provide a more comprehensive and rich theoretical basis for improving China's pension system.

## Data Availability

The data are not publicly available due to restrictions, their containing information that could compromise the privacy of research participants but data can be made available from the Corresponding Author on reasonable requests. The email address is: 17368759281@163.com.
